# A SNP altering the MUC5AC mucin structure is increased in idiopathic pulmonary fibrosis together with the MUC5B SNP

**DOI:** 10.1186/s12931-026-03833-w

**Published:** 2026-07-28

**Authors:** Sergio Trillo-Muyo, Anna Ermund, Brendan Dolan, Levent M. Akyürek, Jesper M. Magnusson, Gunnar C. Hansson

**Affiliations:** 1 Department of Medical Biochemistry and Cell Biology, Institute of Biomedicine, University of Gothenburg, Gothenburg, Sweden; 2 Department of Clinical Pathology, Institute of Biomedicine, Sahlgrenska University Hospital, Gothenburg, Sweden; 3 Department of Respiratory Medicine, Institute of Medicine, Sahlgrenska University Hospital, Gothenburg, Sweden

**Keywords:** Mucin, Mucus, MUC5B, Honeycomb cysts, Pulmonary fibrosis

## Abstract

**Background:**

The gel-forming mucins MUC5B and MUC5AC constitute the main structural components of mucus in the respiratory system where MUC5AC is known to form nets. The MUC5B (rs35705950) Single Nucleotide Polymorphism (SNP) has been shown to increase the risk for Idiopathic Pulmonary Fibrosis (IPF). The MUC5AC SNP rs878913005, common in European populations, results in an Arg1201Trp substitution, resulting in more stabilized secreted mucin nets. Here we investigate the association of rs878913005 with the development of IPF and the presence of the two mucins in IPF diagnostic honeycomb cysts.

**Methods:**

We compared patient groups using whole genome sequences from the UK-Biobank for the SNPs in MUC5AC and MUC5B mucin genes. Lung tissue from IPF patients were immunostained for these mucins.

**Results:**

The MUC5AC SNP shows a significant 1.49x increased frequency in IPF patients relative to controls (*p* < 0.0001) and an odds ratio of 2.09. The well-known IPF-associated SNP in the MUC5B promoter (rs35705950) has a stronger linkage to IPF when combined with the MUC5AC SNP. These two SNPs are located on the same chromosome and are in linkage disequilibrium (r^2^ = 0.10 in controls and up to r^2^ = 0.17 in IPF). Microscopic honeycomb cysts, typical for IPF, are filled with both the MUC5B and MUC5AC mucins with MUC5AC associated to the cysts’ epithelial surface.

**Conclusion:**

A genetic variant with more stable MUC5AC nets in peripheral thin airways may increase the risk of IPF.

**Supplementary Information:**

The online version contains supplementary material available at 10.1186/s12931-026-03833-w.

## Background

Idiopathic pulmonary fibrosis (IPF) is a severe lung disorder with a fast disease progression and poor outcome [1]. IPF is characterized by fibroblast and extracellular matrix expansion where the normal broncho-alveolar architecture is destroyed causing disrupted gas exchange. Whereas first-generation anti-fibrotic therapies have had modest effects, improved understanding of how fibrotic lesions develop are essential for developing therapies that slow progression [2]. However, novel therapies that could prevent disease initiation require understanding of IPF pathogenesis.

The clearance of inhaled foreign material by mucociliary transport is essential for respiratory health. The airway mucus is formed from two secreted mucins, MUC5B and MUC5AC, that both form linear covalent polymers through their N-terminal von Willebrand D3 (VWD3) assemblies and C-terminal cystine-knot domains [3]. This is why the MUC5B mucin polymers form linear bundled strands in the submucosal glands [4]. However, why electron microscopy of MUC5AC show net-like structures was not understood [5]. In our recent study, the molecular structure of MUC5AC VWD3 assembly revealed that these interact non-covalently to form tetramers [6]. Such tetramers will spontaneously result in net-like structures, explaining the observed electron microscopy pictures [5]. These net-like structures efficiently trap microorganisms and facilitate mucociliary clearance [7]. However, MUC5AC is also transiently attached to the epithelium where it controls mucus movement [8, 9] and in chronic diseases, like asthma and COPD, MUC5AC tethering causes mucus retention [10, 11]. IPF is also characterised by mucus accumulation where the honeycomb cysts are filled with mucus [12, 13], although the mechanism linking mucus to cyst formation and IPF is not understood.

An association between a SNP in the *MUC5B* promoter (rs35705950) and IPF was identified over 10 years ago [14]. This association has been confirmed in several studies with this risk allele present in approximately 60% of IPF patients [15]. The presence of such a single T allele increases the risk of IPF 6-fold, whereas two T alleles lead to a 20-fold increase [16]. Studies of the functional role of this SNP have shown that the T allele leads to increased expression levels of *MUC5B* mRNA [17]. Intriguingly, this MUC5B-IPF association is absent in Asian populations [18]. In the healthy lung the terminal airways normally lack expression of the secreted gel-forming mucins MUC5B and MUC5AC [19]. IPF dysplasia of these airways results in the formation of a pseudostratified epithelium with aberrant expression of MUC5B which in turn results in expression of MUC5AC as suggested in an IPF model [20]. This altered tissue architecture displays defective mucociliary clearance despite the mucin production and presence of cilia [13, 21].

During our recent structural studies of the MUC5AC mucin, we observed the presence of amino acid variants of the MUC5AC VWD3 domain assembly at the surface responsible for tetramerization and net formation [6]. These variants were based on two SNPs with identity numbers rs878913005 and rs36189285. The rs878913005 SNP results in an arginine (Arg) to tryptophan (Trp) substitution in the interaction surfaces that cause a stronger interaction of two dimers to give more stable MUC5AC nets [6]. This SNP variant is present in 18.6% of the European population, but only 0.6% of the Asian population where the second SNP (rs36189285) is more common (12–16%) (Genome Aggregation Database, GnomAD). These SNP variants have not been analysed previously using SNP arrays, as relevant sequences have not been included in the array design. The reason for this is their localization close to a highly repetitive and variable region that previously were poorly sequenced and annotated.

The UK-Biobank contains a high number of individuals with corresponding disease and medical information, most of whom have been whole-genome sequenced. Given that rs878913005 strengthens MUC5AC net formation [6], and the close proximity of MUC5AC and MUC5B on chromosome 11, we hypothesized that this variant might contribute to IPF risk—particularly if co-inherited with the MUC5B promoter SNP. Here we investigate the association of rs878913005 with IPF using whole-genome sequencing data from the UK-Biobank and characterize the distribution of MUC5B and MUC5AC mucins in honeycomb cysts from IPF lungs.

## Methods

### UK-Biobank patient cohorts

Control and patient cohorts were defined using the UK-Biobank Research Analysis Platform (DNAnexus). All ethical considerations are covered within the UK-Biobank permissions. Non-Caucasians and participants with whole genome sequence coverage < 95% were excluded. Four patient cohorts were defined, participants with Hospital Diagnosis Records (HDR) including the International Statistical Classification of Diseases and Related Health Problems 10th Revision (ICD10) annotation J84.1: Other interstitial pulmonary diseases with fibrosis (cohort ‘PF’), participants with underlying (primary) cause of death ICD10 J84.1 (cohort ‘PF-Death’), IPF doctor diagnosed participants (cohort ‘IPF’) and participants included both in PF and IPF cohorts (‘PF&IPF’). The participants with a negative diagnosis of IPF excluding those diagnosed with other interstitial pulmonary diseases with fibrosis (HDR ICD10: J84.1) composed the initial control group (*n* = 88,977). The control group was age-, sex-, and smoking status-matched using the ‘IPF’ cohort as a reference (*n* = 27,936).

### MUC5B and MUC5AC allelic variants

The genetic information regarding chromosome 11 contained in the genomic variant call format (gVCF) files was extracted from all cohorts. Annotations in GRCh38 position 1,177,533 (MUC5AC gene, rs36189285, nucleotide G > A, amino acid Arg996Gln), 1,180,138 (*MUC5AC* gene, rs878913005, nucleotide C > T, amino acid Arg1201Trp) and 1,219,991 (*MUC5B* promoter, rs35705950, nucleotide G > T, A) were analysed and participants were classified as SNP carriers (heterozygous and homozygous) or WT (wild type). Both explicit variant records and gVCF reference blocks were considered, allowing high-quality genotypes to be distinguished from missing or no-call genotypes. Genotype-level quality control was performed for the analysed variants. Genotype calls were retained if genotype quality was ≥ 20 and read depth was ≥ 10. Participants with low-quality genotype information in any analysed variant were excluded from the final analyses. The database used was from November 30, 2023.

### PCR detection of rs35705950, rs878913005, and rs36189285 SNPs

3 × 10 μm sections were cut from paraffin embedded formalin fixed lung tissue and collected in 2 ml microcentrifuge tubes. DNA was then isolated using QIAamp DNA FFPE Advanced Kit (Qiagen) according to manufacturer’s instructions. Following isolation DNA was quantified using a Nanodrop 2000 spectrophotometer (Thermo Fisher Scientific, RRID: SCR_018042) and diluted to 10 ng/µl in TE buffer. DNA was stored at -20 °C until use.

Specific assays were designed to identify reference and alternate alleles for the rs35705950, rs878913005 and rs36189285 SNPs using the following primers; (1) rs35705950 allele primer 1: /rhAmp-FAM/TTCCTTTATCTTCTGTTTTCAGCGrCCTTC /GT4/, allele primer 2: /rhAmp-Yakima Yellow/ CTCCTTTATCTTCTGTTTTCAGCGrCCTTC /GT4/ and locus primer: GCGTTTGCTCAGCGTCTTTCAArGAGTT/GT3/ and (2) rs878913005 allele primer 1: /rhAmp-FAM/ACCTTCCAGGCCCCGrGACGT/GT3/, allele primer 2: /rhAmp-Yakima Yellow/ACCTTCCAGGCCCCArGACGT/GT3/ and locus primer: GCAGCTCTGTTCTGCGACTACTArCAACC/GT3/ and (3) rs36189285 allele primer 1: /rhAmp-FAM/GGTAGATGCCCATCTGCCrGGATG /GT2/, allele primer 2: /rhAmp-Yakima Yellow/AGGTAGATGCCCATCTGCTrGGATG /GT2/ and locus primer: GCTGAAGCTAAGCCATGGGAAGrGTGGA/GT2/. Control plasmids were synthesised containing the DNA sequence − 200 to + 200 base pairs either side of the reference or alternate alleles for the rs35705950, rs878913005 and rs36189285. Primers were synthesized by Integrated DNA Technologies and control plasmids were synthesised by Thermo Fisher Scientific.

PCR detection of rs35705950, rs878913005 and rs36189285 SNPs was performed using rhAmp™ SNP Genotyping (Integrated DNA Technologies) according to manufacturer’s protocol using a CFX96 real-time PCR system (Bio-Rad). The following cycling conditions were used; enzyme activation for 10 min at 95 °C followed 40 cycles of 95 °C for 10 s, 60 °C for 30 s and 68 °C for 20 s. Following each cycle, emission of fluorescent signal was detected in the FAM and VIC/Yakima Yellow channels. Reactions contained 20 ng of template DNA or 500 copies of control plasmid. Amplification data was analysed using CFX Manager Software (v3.1; Bio-Rad).

### Human tissue samples

The studies were conducted in accordance with the Declaration of Helsinki. All alive subjects at the time of the study provided informed written consent prior to inclusion. Transbronchial cryo-biopsies were acquired during routine bronchoscopy for diagnosis of parenchymal/interstitial lung disease at the Sahlgrenska University Hospital, Gothenburg, Sweden. The resulting cryo-biopsy was larger than a traditional forceps biopsy [22]. The frozen biopsy was submerged in saline to release the biopsy, fixed in 10% neutral buffered formalin, transferred to 70% ethanol, and embedded in paraffin (ethical permission 543 − 11).

Lung samples from IPF patients were acquired during resection of diseased lungs at transplantation (ethical permission 2020–03693). Control lung samples were acquired from patients undergoing resection for lung adenocarcinoma (ethical permission 543 − 11). Pieces of lung parenchyma were fixed in 10% neutral buffered formalin, transferred to 70% ethanol and embedded in paraffin at the Sahlgrenska University Hospital. Sections of 4 μm were cut and mounted on SuperFrost Plus slides (Cat# 631–9483, VWR, Avantor, Radnor Township, PA). Researchers had access to medical records.

### MUC5B antibody

The MUC5B-D3 and MUC5AC-D3 assemblies were heterologously expressed in CHO-Lec 3.2.8.1-S cells and purified as described in [23] and [6]. The purified MUC5B protein was immobilized on agarose resin by affinity- chromatography using the AminoLink™ Immobilization Kit (Thermo Fisher Scientific). MUC5B polyclonal antibodies were produced in rabbit as detailed in [23]. The antiserum was loaded onto a HiTrap Protein A HP 5 ml column (GE Healthcare) equilibrated with 20 mM sodium phosphate pH 8. The antibodies were eluted in 0.1 M citric acid pH 3 and neutralized by 1 M Tris-HCl pH 9. The Protein A purified serum was loaded onto the AminoLink-MUC5B column equilibrated with PBS pH 7.2 and incubated for 1 h, washed with 6 column volumes PBS, and bound antibodies eluted with 0.1 M citric acid pH 3, neutralized with 1 M Tris-HCl pH 9, and loaded onto an AminoLink-MUC5AC column. After 1 h, the unbound final anti-MUC5B antiserum was washed out with PBS pH 7.2.

### Histological staining

Sections were stained with Harris hematoxylin (Cat# 01800, Histolab, Askim, Sweden) and Eosin Y (Cat# 01650, Histolab, Askim, Sweden) according to standard protocols. For visualization of connective tissue, a Masson-Goldner staining kit was used (Cat# 1.00485, Sigma-Aldrich/Merck). Nuclei were stained with Weigert´s iron hematoxylin (Cat# 1,15973, Sigma-Aldrich/Merck, Darmstadt, Germany). Sections were mounted with borosilicate cover glass (Cat# 831 − 0137, VWR international, Radnor, PA) using DPX mounting medium (Cat# 06522, Sigma-Aldrich/Merck).

### Immunofluorescent staining of histological sections

Sections were baked on the slides at 60 °C for 2 h, dewaxed using xylene and hydrated. Antigen heat-induced epitope retrieval was performed with 10 mM citrate buffer pH 6.0 at 100 °C for 20 min and then room temperature for another 20 min. Sections were washed in PBS, a barrier was drawn with an ImmEdge^®^ Hydrophobic Barrier PAP Pen (H-4000, Vector laboratories, Newark, CA). Unspecific epitopes were blocked with 3% donkey serum in Tris-buffered saline and sections were permeabilized with 0.1% Triton X-100. Stainings were performed with sequential incubation with the described purified rabbit anti-human MUC5B antibody (1:200) in block solution [23] overnight at 4 °C, monoclonal mouse anti-human MUC5AC, clone 45M1 (1:200) [24] in block solution over night at 4 °C (Cat# ab3649, Abcam, Cambridge, UK, RRID: AB_2146844), and purified goat anti-human EpCAM/TROP-1 (1:200) in block solution over night at 4 °C (Cat# AF960, R and D Systems/Biotechne, Minneapolis, MN, RRID: AB_355745). Secondary antibodies were polyclonal donkey anti-goat Alexa Fluor 488 (Cat# A-11055, Thermo Fisher Scientific, Waltham, MA, RRID: AB_2534102), polyclonal donkey anti-rabbit Alexa Fluor 555 (Cat# A-31572, Thermo Fisher Scientific, RRID: AB_162543), polyclonal donkey, anti-mouse Alexa Fluor 647 (Cat# A-31571, Thermo Fisher Scientific, RRID: AB_162542). Nuclei were stained with Hoechst 34,580 (Cat# 565877, BD Biosciences, Franklin Lakes, NJ, RRID: AB_2869723). Sections were mounted using ProLong™ Gold Antifade Mountant (Cat# P36930, Thermo Fischer Scientific) and borosilicate glass coverslips (Cat# 831 − 0137, VWR international, Radnor, PA).

### Imaging

Sections stained with histological stains (H&E, Masson-Goldner) were scanned using a NanoZoomer-SQ digital slide scanner (Cat# C13140-01, Hamamatsu Photonics, Shizuoka, Japan, RRID: SCR_023763) and NDP.view2 Image viewing software (Hamamatsu Photonics, Shizuoka, Japan, RRID: SCR_025177). Epi-fluorescent images were acquired using a Nikon Eclipse Ci-E microscope (Nikon instruments, Tokyo, Japan), a Nikon microscope camera DS-Fi3 (RRID: SCR_018857) and NIS-Elements D software version 5.02.03 (Nikon instruments, RRID: SCR_014329). High resolution images were acquired using ZEISS ZEN Microscopy Software (Carl Zeiss, Oberkochen, Germany, RRID: SCR_013672) on the ZEISS LSM900 with Airyscan 2 (Carl Zeiss, Oberkochen, Germany, RRID: SCR_022263) and processed with Imaris software version 9.5.0 (Oxford Instruments, Abingdon, UK, RRID: SCR_007370).

### Statistical analyses

Statistical analyses of disease associations for individual SNPs were performed using the Cochran–Armitage trend test. Associations between individual SNPs, combinations of SNPs, and disease status were analysed using Pearson’s chi-square test. Correction for multiple testing was applied using the Benjamini–Hochberg false discovery rate (FDR) procedure, considering 20 association tests and, separately, 5 linkage disequilibrium tests. Statistical significance was defined as a q-value < 0.05 after FDR correction. Adjusted *p*-values (q-values) are reported in Supplementary Table S1. All statistical analyses were performed using IBM^®^ SPSS Statistics (RRID: SCR_016479).

## Results

### Patients

**Fig. 1:**
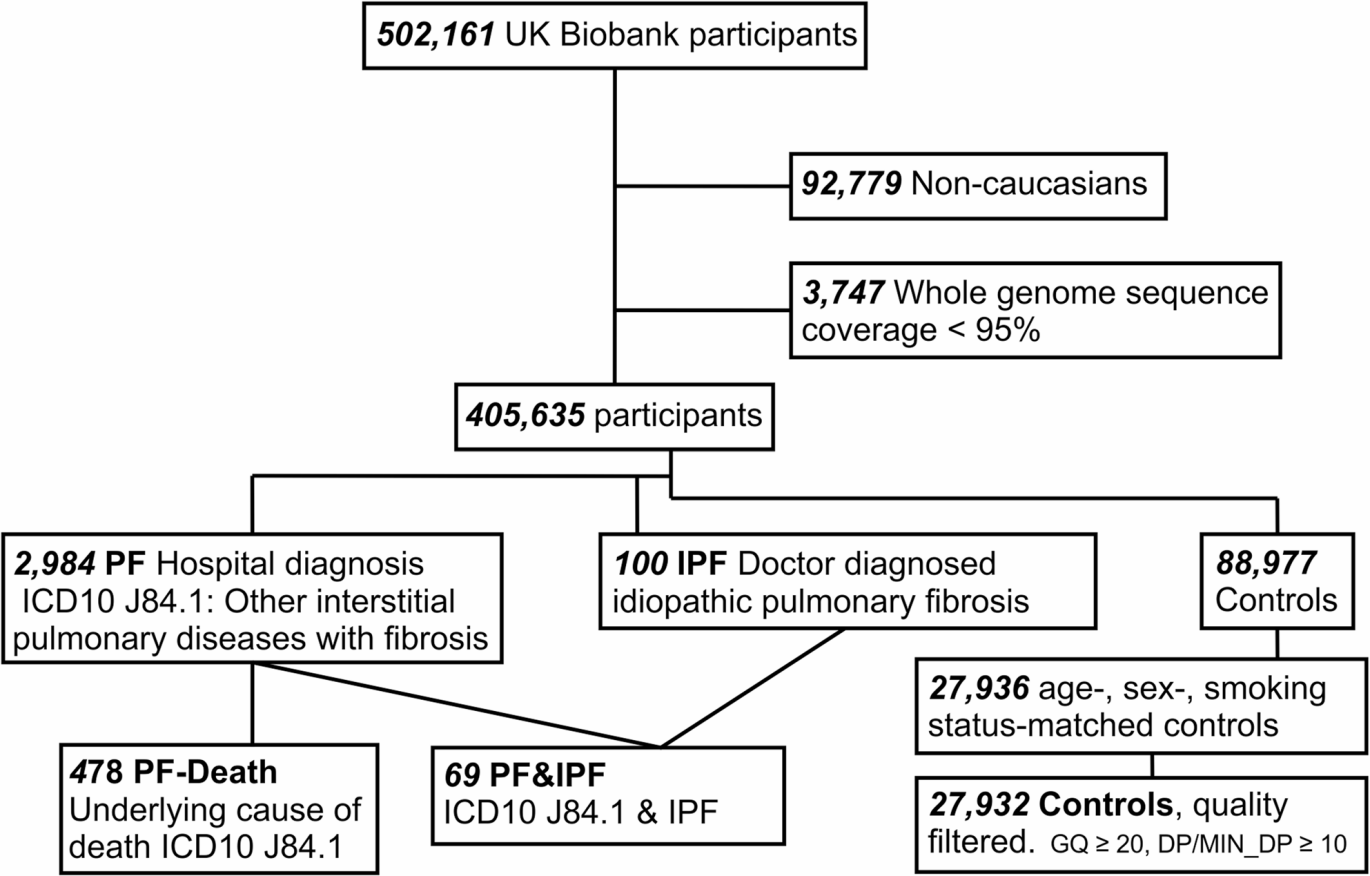
Study flow diagram of UK-Biobank patients analysed. The numbers refer to number of participants in each group. The groups analysed are marked; PF (J84.1), PF-Death (J84.1 and Dead), IPF (doctor diagnosed IPF), and PF&IPF (J84.1 and IPF), and Controls. GQ, genotype quality; DP, read depth; MIN_DP, minimum read depth across a gVCF reference block. Genotype-level quality control was applied to the analysed SNP positions in all participant groups

The coupling of the MUC5AC rs878913005 and rs36189285 ‘Asian’ SNP to disease was studied in the complete genomes of individuals included in the UK-Biobank. The number of individuals with the rs36189285 SNP was low (two persons) and these were not further analysed. The genotypes were compared to the available medical information. The nature of IPF makes genotypic association studies complex due to elusive IPF diagnosis. The ICD10 classification does not include a specific diagnosis for IPF, it is included in the miscellaneous group, J84.1: ‘other interstitial pulmonary diseases with fibrosis’. The UK-Biobank participants with HDR classified as J84.1 constituted the largest cohort in the present study and were labelled ‘PF’ for pulmonary fibrosis (Fig. 1, ‘PF’, *n* = 2,984). As IPF is a lethal and fast-progressing disease, the cause of death in the J84.1 cohort allowed us to more accurately identify the patients with IPF included in this group, which were named ‘PF-Death’ (Fig. 1, ‘PF-Death’, *n* = 478). The UK-Biobank includes a self-reported data field (#22135) where 121,270 participants were asked if a doctor had ever told them that they had IPF. This cohort named ‘IPF’ (Fig. 1, ‘IPF’, *n* = 100) is smaller and has the limitations associated with self-reporting, but it is better defined than those above (‘PF’ and ‘PF-Death’) as it refers specifically to IPF. Consequently, this group was used as reference for assembling an age-, sex-, and smoking status-matched control group (Fig. 1, ‘Controls’, *n* = 27,936). Individuals classified in both the ‘PF’ and ‘IPF’ cohorts gave a well-defined, but small, confirmed IPF group named ‘PF&IPF’ (Fig. 1, ‘PF&IPF’, *n* = 69). The structure of the patient cohorts analysed in this study are summarised in Fig. 1.

### Association between the MUC5AC rs878913005 SNP and IPF

**Fig. 2:**
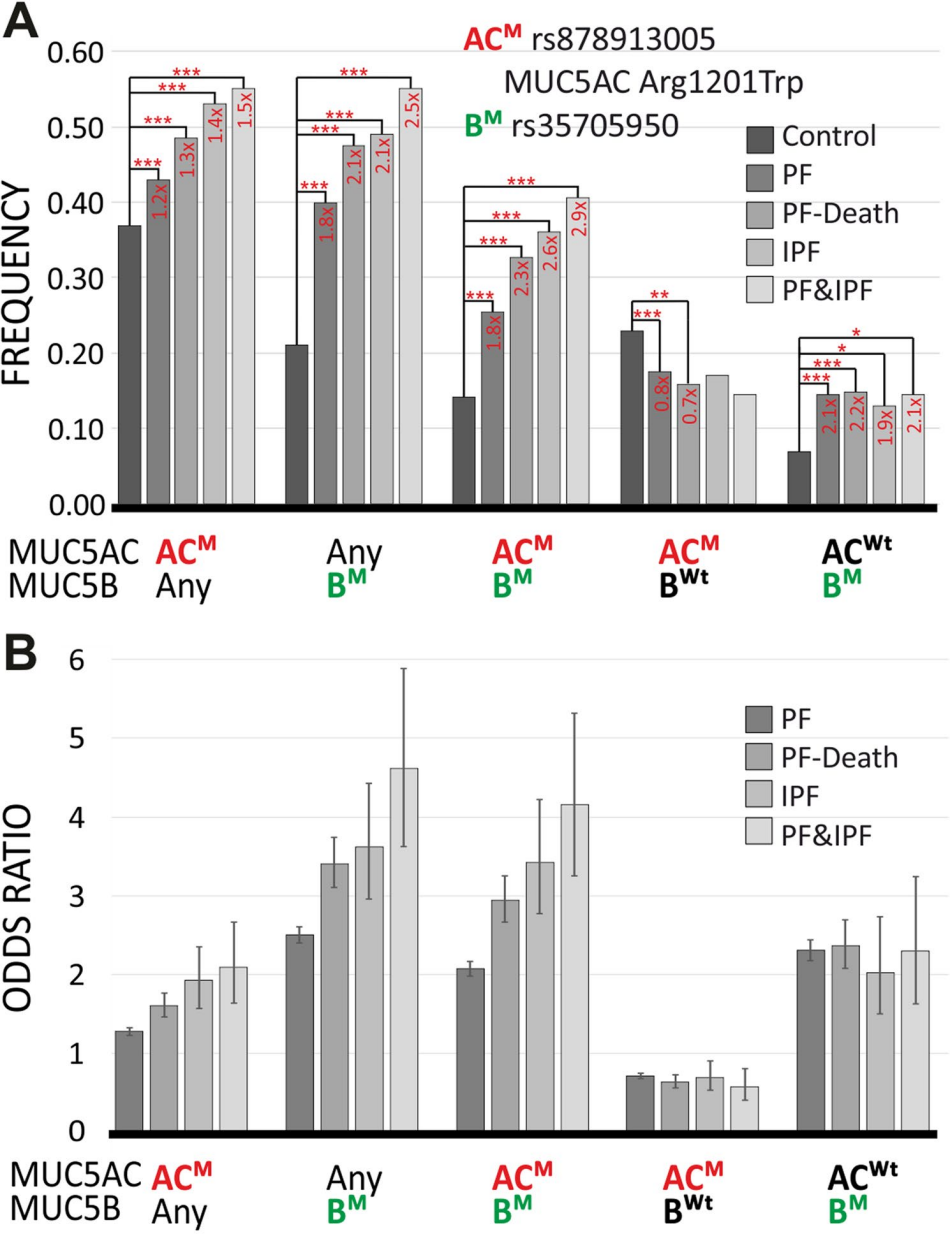
Analysis of the association between mucin gene SNPs and IPF. **A** Frequency of the MUC5AC SNP (rs878913005) in control and patient cohorts. Numbers in bars indicate fold-frequency change compared to the control cohort, when differences are statistically significant. * q < 0.02, ** q < 0.0005 and, *** q < 0.00005, using the Cochran–Armitage trend test and the Pearson’s chi-square test with correction for multiple testing with FDR. AC^WT^ indicates wildtype MUC5AC, and AC^M^ indicates variant MUC5AC (rs878913005). B^Wt^ indicates wildtype MUC5B, and B^M^ indicates variant MUC5B (rs35705950). **B** Odds ratio calculated for the different patient cohorts. OR of developing IPF when harbouring SNPs in the gel-forming mucins (individual SNPs, combination of both and excluding the other) to the odds of developing IPF without carrying the SNPs. Error bars represent the 95% confidence interval

The MUC5AC SNP rs878913005 was searched for in all cohorts, with no missing calls identified. Four control participants were excluded for not meeting the genotype quality and read depth control criteria. Although the PF&IPF cohort was relatively small (*n* = 69), 38 of these patients (55.07%, Supplementary Table S1) had at least one allele harbouring the rs878913005 SNP. This is compared to the 36.95% (*n* = 10,323) of the control group (*n* = 27,932, Supplementary Table S1). It means that IPF patients showed a significant 1.49-fold increase in SNP frequency (Cochran–Armitage’s trend test, *p* = 0.000041, Fig. 2A, AC^M^/Any, Supplementary Table S1), and an odds ratio (OR) of 2.09 (95% confidence interval: 1.64–2.66, Fig. 2B, AC^M^/Any). The other three cohorts also showed an association between the SNP and the disease. The associations were weaker in terms of OR, likely due to lower accuracy of the IPF diagnosis and less defined cohorts (PF&IPF > IPF>PF-Death > PF), but statistically more significant due to the higher number of participants (Fig. 2, Supplementary Table S1).

The well-documented IPF-associated SNP rs35705950 affecting the MUC5B mucin promoter region was analysed in the same UK-Biobank cohorts, also with no missing calls identified [14]. As expected, a significant 2.49-fold (*p* < 0.00001) increase in frequency of this SNP in the PF&IPF cohort was found (Fig. 2A, B^M^/Any, Supplementary Table S1) and an OR of 4.59 (95% confidence interval: 3.61–5.85, Fig. 2B, B^M^/Any). The statistical association through the different cohorts followed the same trend as for the MUC5AC SNP (Fig. 2, Supplementary Table S1). Both analysed SNPs were in Hardy-Weinberg equilibrium in the control group, rs878913005 *p* = 0.756, rs35705950, *p* = 0.566.

Interestingly, when both SNPs were analysed in relation to each other, the association between SNPs and disease was stronger when both SNPs appeared in the same individual. The fold-frequency increase of the two SNPs together in IPF patients (PF&IPF cohort) is higher (2.88-fold increase, *p* < 0.00001, Fig. 2A, AC^M^/B^M^) than when only the MUC5B SNP is present in combination with WT for MUC5AC (2.11-fold increase, *p* = 0.01228, Fig. 2a, AC^WT^/B^M^). When only the MUC5B SNP was present together with WT MUC5AC, the odds ratio was reduced by 44% from 4.12 to 2.30 (Fig. 2B, AC^Wt^/B^M^) suggesting that the well-studied MUC5B SNP variant in isolation might be of lower importance in the absence of the MUC5AC SNP (Fig. 2B, AC^M^/B^Wt^, Supplementary Table S1). However, the disease association of the MUC5AC SNP is even more dependent on the presence MUC5B SNP as the odds ratio of the MUC5AC SNP in isolation is 0.57 (Fig. 2B, AC^M^/B^Wt^, Supplementary Table S1). Surprisingly, when the MUC5B SNP is present in the absence of the MUC5AC SNP the trend in frequency increase between groups disappear (Fig. 2A, AC^Wt^/B^M^). This could, of course, be explained by the loss of accuracy due to lower frequencies in the smaller cohorts.

**Fig. 3:**
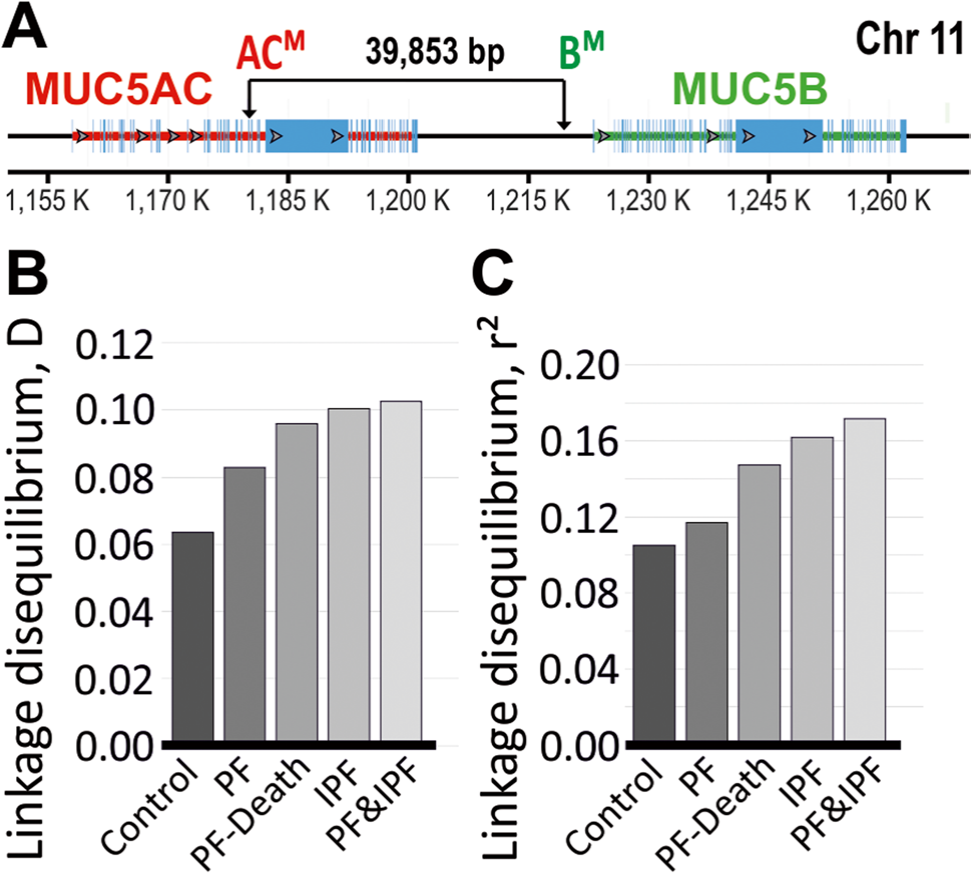
Linkage disequilibrium between the SNPs rs878913005 and rs35705950. **A** Genomic organization of the MUC5AC and MUC5B gene locus in chromosome 11. **B** Difference between the observed and expected haplotype frequencies, D, between MUC5AC rs878913005 and MUC5B promoter rs35705950 in the different cohorts. **C** Linkage disequilibrium between MUC5AC rs878913005 and MUC5B promoter rs35705950 in the different cohorts expressed in terms of the squared correlation (r^2^)

The MUC5AC and MUC5B mucins are located close together on chromosome 11 with 39,853 nucleotides between the two IPF-associated SNPs (Fig. 3A). A genetic link between the two is therefore possible. Analysis for linkage disequilibrium revealed a difference between the observed and expected haplotype frequencies (D) of 0.064 for rs878913005 (MUC5AC) and rs35705950 (MUC5B) in the control group (Fig. 3B, Supplementary Table S1). However, the linkage disequilibrium between the two SNPs increases to 0.102 in the PF&IPF cohort. Consequently, the frequency of having both SNPs increases from the expected 0.077 to the observed 0.141 in the control group, while it increases from 0.303 to 0.405 in the IPF group, meaning that the probability of presenting both SNPs is almost 3 times higher in IPF patients. The r^2^ value rises from 0.1063 in the control group to 0.1716 in PF&IPF (Fig. 3C, Supplementary Table S1). Control of the false discovery rate using the Benjamini-Hochberg procedure did not alter the interpretation of the associations. Linkage disequilibrium tests between the two SNPs remained significant after FDR correction.

Together the results suggest that the amino acid change (Arg1201Trp) in MUC5AC and increased MUC5B promoter activity due to the rs878913005 and rs35705950 SNPs, respectively, are associated with an increased risk of IPF. It is possible that both SNPs often are present together, likely on the same chromosome. This may help explain the decreased relation to disease when associated with the presence of a single MUC5AC or MUC5B SNP in the different cohorts.

### Mucins and IPF

**Fig. 4:**
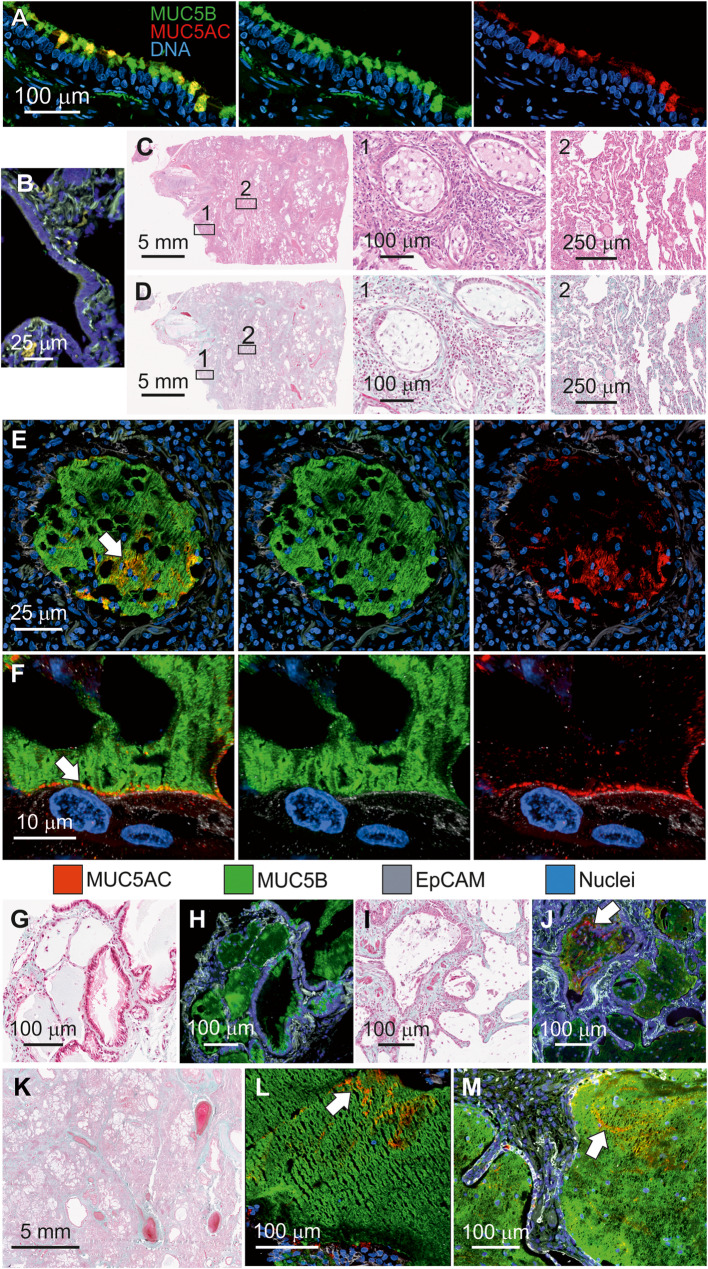
The MUC5AC and MUC5B mucins are accumulated in IPF honeycomb cysts. **A** Immunofluorescent staining of a bronchiole from IPF patient #3 displaying goblet cell hyperplasia with goblet cells containing both mucins, MUC5AC (red) and MUC5B (green). Nuclei are blue. **B** Immunofluorescent staining for mucins MUC5AC (red), MUC5B (green) and epithelial marker EpCAM (grey) in a section from non-IPF patient #7 demonstrates respiratory bronchioles without goblet cells or mucins. **C** H&E-stained lung tissue from IPF patient #2. Insets highlight the presence of (1) microscopic honeycomb cysts and (2) alveolar wall thickening. **D** Masson-Goldner-stained lung tissue from IPF patient #2. Insets highlight the presence of fibrosis (1) around microscopic honeycomb cysts and (2) in alveolar regions. **E** High-resolution micrograph of honeycomb cyst from patient #2 stained for MUC5AC, MUC5B, EpCAM, and DNA. **F** High-resolution micrograph of epithelial surface from a honeycomb cyst from patient #2 stained for MUC5AC, MUC5B, EpCAM, and DNA. **G** Masson-Goldner-stained lung tissue from IPF patient #1 and similar section in **H** epifluorescence image of honeycomb cyst stained for MUC5AC, MUC5B, EpCAM, and DNA. Only a small amount of tissue was available from this patient #1 as this is from a transbronchial cryo-biopsy. **I** Masson-Goldner-stained lung tissue from IPF patient #5. **J** Epifluorescence image of honeycomb cyst from patient #5 stained for MUC5AC, MUC5B, EpCAM, and DNA. **K** Masson-Goldner-stained lung tissue from IPF patient #4. **L** High-resolution micrograph of honeycomb cyst from patient #4 stained for MUC5AC, MUC5B, EpCAM, and DNA. **M** Epifluorescence image of honeycomb cyst from patient #3 stained for MUC5AC, MUC5B, EpCAM, and DNA. White arrows in E, F, J, L and M point to MUC5AC in honeycomb cysts where the suggested MUC5AC sheets have been cut transversally in F and M and in parallel in E. The suggested sheets in F and M were observed in adjacent section arguing for their suggested flat nature

The MUC5B and MUC5AC mucins are both normally absent from the smallest human airways, terminal and respiratory bronchioles [19, 25, 26]. To study the presence of MUC5B and MUC5AC, tissues were obtained from lungs removed following transplant of IPF patients, cryo-biopsies, and from non-cancer tissues resected for lung adenocarcinoma. The MUC5AC and MUC5B SNP genotypes were determined (Supplementary Table S3). In IPF patients, goblet cell hyperplasia can be observed in peripheral bronchioles. The goblet cells are filled with both the MUC5AC and MUC5B mucins (Fig. 4A). In contrast, the airway surface epithelium of peripheral bronchioles in control patients were without goblet cells or cells containing mucins (Fig. 4B), as has also been observed previously [19, 25, 26]. Fibrosis or honeycomb cysts were not observed in control patient lung tissue (not shown). As expected, the tissue in IPF lungs show severe destruction and fibrosis as shown with H&E staining (Fig. 4C) or Masson-Goldner (Fig. 4D) including microscopic honeycomb cysts characteristic for IPF (Fig. 4CD, Inset 1: honeycomb cyst, Inset 2: fibrosis) [12, 27]. These cysts are filled with mucus where the MUC5B mucins predominates [13], but the MUC5AC mucin is also observed (Fig. 4E). The MUC5B mucin fills out the honey comb cysts with a diffuse staining pattern and detailed studies show that the MUC5AC mucin appear as sheets and is localized at the outer rim of the cysts, sometimes in direct contact with the cyst epithelium (Fig. 4D). Tissues from additional four IPF patients in Figs. 4G-M show MUC5B filled honeycomb cysts with MUC5AC sheets (except patient #1, Fig. 4GH from a cryo biopsy). Interestingly, the MUC5AC staining suggests that this mucin forms continuous sheets or layers lining the epithelium, rather than a diffuse pattern staining as for MUC5B, suggesting the formation of higher order structures as suggested from the net-like MUC5AC mucin sheets [6].

## Discussion

Alveolar scarring and fibrosis are the hallmarks of IPF, yet how the disease is initiated and why honeycomb cysts are characteristic remain unclear. Understanding these early pathogenic events is critical for developing curative therapies. Our findings identify the MUC5AC rs878913005 SNP as a novel genetic risk factor for IPF, potentially helping to explain disease initiation. This variant strengthens MUC5AC tetramerization, creating more stable mucin nets anchoring the mucus, something that might impair mucociliary clearance in distal airways. Together with the well-established MUC5B promoter SNP, these findings indicate that mucus dysfunction, both overproduction and impaired clearance, may trigger the immune cell infiltration and fibroblast proliferation that culminates in IPF, particularly when genetically susceptible individuals encounter environmental insults.

**Fig. 5:**
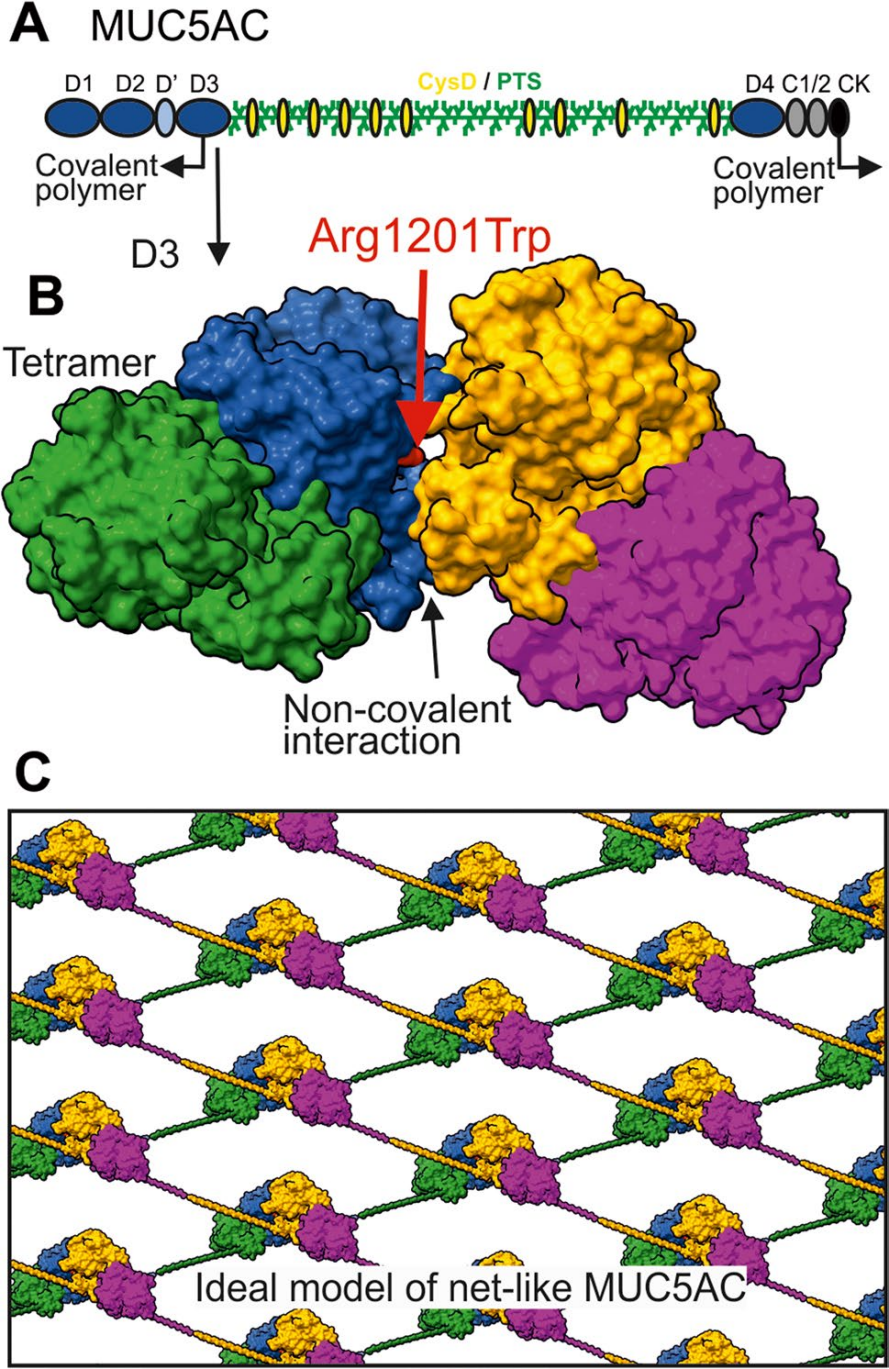
Molecular structure of the MUC5AC mucin VWD3 assembly and its interactions leading to net-like formations. **A** The MUC5AC mucin domain organization with the sites for the polymer formation by N- to N-terminal and C- to C-terminal covalent bonds. Abbreviations correspond to D1/2/3/4: von-Willebrand D assembly, D’: von-Willebrand D’ domain, CysD: cysteine rich domain, PTS: mucin domain, C1/2: von-Willebrand C domain and CK: cystine-knot. The site for non-covalent interactions at the von Willebrand D3 (D3) assembly are indicated (arrow). **B** Molecular structure of the tetramer formed by non-covalent interaction by two D3 assemblies with the localization of the amino acid substitution (Arg1201Trp, red) as a result of the SNP rs878913005. **C** Proposed ideal model of how MUC5AC can form net-like structures by non-covalent interactions. Structures and models generated from previously published data [6]

The respiratory system has two mucins, the MUC5B and MUC5AC, that both are linear polymers linked together by covalent disulfide bonds in their von Willebrand D3 (D3) and cystine-knot (CK) domains (Fig. 5A). The MUC5AC mucin has additional interaction sites as microscopy images show net-like structures [5]. The nature of this interaction was recently explained by molecular structural studies using cryo-electron microscopy [6]. Two MUC5AC polymers interact non-covalently by the identical surface formed from two different VWD3 dimer assemblies (Fig. 5B) [6]. Such an interaction will allow the polymers to form net-like structures as illustrated in an ideal model (Fig. 5C). Net-like formations have a high capacity to trap microorganisms and other foreign material, a key step in cleaning the airways [7].

We observed a significant amino acid variation at the interaction surface forming tetramers caused by a SNP frequent in European populations [6]. This SNP has the identification number rs878913005 and encodes the replacement of a positively charged amino acid, arginine, to a neutral amino acid, tryptophan, giving the Arg1201Trp substitution [6]. The localization of this amino acid substitution is marked in Fig. 5B. The Arg1201Trp substitution results in a stronger interaction, increased stabilisation of the tetramers, and more stable nets [6]. This MUC5AC rs878913005 SNP has not been extensively studied, though it was recently linked to inflammatory dysregulation in keratoconus [28]. Using whole-genome sequences from the UK-Biobank, we found that the strongest disease association for this SNP was with IPF. The SNP was present in 55.07% of our most well-defined IPF cohort versus 36.95% in controls—a 1.49-fold increase (OR = 2.09, 95% CI: 1.64–2.66). While IPF diagnoses in the UK-Biobank have inherent limitations, our replication of the well-established MUC5B SNP association (OR = 4.62) validates our cohort stratification [14].

The second MUC5AC SNP that is common in Asian populations, rs36189285, might also affect MUC5AC tetramerization and net-formation [6]. However, this was too rare in our UK-Biobank cohort for analysis. The rs36189285 SNP is frequent among Koreans (33%) [29], but a recent genomic study of IPF patients did not observe any relation between this SNP and IPF in this population [30].

The association between the MUC5B promoter SNP and IPF has been clearly identified in previous studies [14] and in animal models where overexpression of MUC5B and lack of MUC5B have been shown to result in increased and decreased disease severity in experimental IPF models [21]. Despite previous studies on the importance of MUC5B in the development of IPF these have been somewhat puzzling as the MUC5B promoter SNP was not associated with IPF in Asian populations [18]. This may now be explained by the identified genetic linkage between the rs35705950 (MUC5B) and rs878913005 (MUC5AC) (r²=0.10 in controls, 0.17 in IPF) SNPs. Critically, rs878913005 is rare in Asian populations (0.6% vs. 18.6% in Europeans) [6]. The low odds ratio for the MUC5AC SNP rs878913005 in isolation may be explained by aberrant MUC5B expression and epithelial dysplasia being required for expression of MUC5AC in the distal airways. When both SNPs occur together, the odds ratio for IPF increases substantially (OR = 4.16), while either SNP in isolation shows weaker association across our cohorts. Therefore, it is likely that the strong association between the previously identified MUC5B (rs35705950) SNP and IPF is partly due to its linkage to the presence of the MUC5AC (rs878913005) SNP. The linkage of the two SNPs could maybe be explained by both being present as successive genes on the same chromosome. The reason for the relation of the two SNPs is not understood today, but it could have been an evolutionary advantage for young individuals to have these SNPs as they might improve handling of lung infections by increased levels (MUC5B) and stronger (MUC5AC) mucin-nets. However, the late-in-life appearance of IPF, which we now observe, would not have posed an evolutionary disadvantage.

Along the extensive damage and fibrosis of the peripheral airways, IPF is characterised by the presence of microscopic honeycomb cysts [12]. The honeycomb cysts are filled with mucus but can often appear empty due to technical difficulties in preserving mucus [12, 13]. As the honeycomb cysts are mucus filled, mucus accumulation might be involved in the formation of the cysts. MUC5B is the most abundant mucin as has also been described previously [13, 31], but the MUC5AC protein is also present [31]. In the normal healthy lung, there are no gel-forming mucins produced and no goblet cell present in the most distal and peripheral airways [13, 19, 32]. However, both MUC5B and MUC5AC mucins are increased in surface epithelial cells of peripheral IPF airways [13, 33]. Here we show that MUC5AC in the honeycomb cysts is not as diffusely spread out as MUC5B, but rather associated with the luminal surface of the epithelial cells lining the cyst. The net-like structure of secreted MUC5AC results in the spontaneous formation of sheet-like formations associated with the epithelial surface. This may suggest that surface-tethered MUC5AC plays a role in the retention and subsequent accumulation of mucus in the distal airways. This is similar to other lung diseases, as asthma and COPD, where the MUC5AC mucin is increased and tethered to the respiratory tract epithelium where it causes mucus accumulation and retention [10, 11]. Accumulated and attached MUC5AC and MUC5B mucins in peripheral airways could provide a medium for the trapping and accumulation of inhaled particles and bacteria, something that could trigger immune cell infiltration. Mucus retention in peripheral airways could maybe also initiate a fibrotic cascade.

When Lorenzo-Salazar *et al*. deep sequenced three areas around a suggested IPF susceptibility locus, they discovered two SNPs (rs34474233 and rs34815853) that cause an alanine 5353 to lysine amino acid modification in the C-terminal of the MUC5AC mucin [34]. The approach used by these authors did not reach the rs878913005 SNP in the MUC5AC N-terminus. The Ala5353Lys modification in the VWC’ of the MUC5AC C-terminus is likely exposing this new Lys [35]. These IPF-associated SNPs further suggest that mucins could have a role in IPF.

While our findings are limited by the diagnostic heterogeneity inherent to the UK-Biobank database and its restriction to European populations, they establish a foundation for further mechanistic studies. The replication of the MUC5B association and the biological plausibility of our observations support their validity. However, several critical questions remain. Future studies should further clarify how Arg1201Trp affects mucus attachment and clearance using human airway organoids or air-liquid interface cultures. It is important to do longitudinal studies to track mucin expression and mucus accumulation in early-stage IPF to establish temporal relationships. This study should be replicated in large, ethnically diverse, and histologically confirmed IPF cohorts, particularly in Asian populations where rs36189285 predominates. Further investigations should address whether mucolytic or mucus-modulating therapies (e.g., N-acetylcysteine, hypertonic saline, or MUC5AC-targeted antibodies) could prevent disease progression in genetically susceptible individuals. Further mechanistic studies should also define if mucus accumulation can trigger the epithelial-mesenchymal-immune cell crosstalk that drives fibroblast activation. Understanding these early events may identify therapeutic windows for disease interception before the irreversible fibrosis develops, shifting the paradigm from slowing disease progression to its prevention.

## Supplementary Information

Supplementary Material 1: Supplementary Information.

## Data Availability

No datasets were generated or analysed during the current study.
