# Supplementary material for: A SNP altering the MUC5AC mucin structure is increased in idiopathic pulmonary fibrosis together with the MUC5B SNP
Source: Respir Res. 2026 Jul 28;27:304. doi: 10.1186/s12931-026-03833-w (PMC13453088; doi:10.1186/s12931-026-03833-w)
Supplement: Supplementary file 1 [file 12931_2026_3833_MOESM1_ESM.pdf]

SUPPLEMENTARY INFORMATION

SUPPLEMENTAL TABLE S1. Results and statistics, pages 2

SUPPLEMENTAL TABLE S2. UK Biobank patients, pages 3-4

SUPPLEMENTAL TABLE S3. IPF and Control patients used for staining, page 5

SUPPLEMENTARY TABLE S1. MUC5AC (AC) rs878913005 SNP and MUC5B (B) promotor rs35705950 SNPs.

	Controls	Hospital diagnosis record (HDR) ICD10: J84.1	Underlying cause of death ICD10: J84.1	Doctor diagnosed IPF	Doctor diagnosed IPF & HDR J84.1
**Participants**[#]	27,932	2,984	478	100	69
**AC WT (AC^{WT})**[#]	17,602 (63)	1,701 (57)	245 (51)	47 (47)	31 (45)
**AC Mutant (AC^{M})**[#]	10,330 (37)	1,283 (43)	233 (49)	53 (53)	38 (55)
Heterozygots[#]	9,132 (33)	1,100 (37)	198 (41)	41 (41)	28 (41)
Homozygots[#]	1,198 (4)	183 (6)	35 (7)	12 (12)	10 (14)
T*		6,548,760	1,975,392	662,780	545,304
Var(T)*		8.58E+11	1.25E+11	2.57E+10	1.77E+10
SD*		926,308	353,591	160,466	133,204
Z*		7.0697	5.5867	4.1303	4.0937
p-value*		< 0.00001	< 0.00001	0.000036	0.000041
q-value[Δ]		0.000015	0.000015	0.000050	0.000055
RR[+]		1.25	1.61	1.92	2.08
OR[Φ]		1.29	1.62	1.92	2.09
		(1.24 – 1.34)	(1.49 – 1.78)	(1.57 – 2.35)	(1.64 – 2.66)
**B WT (B^{WT})**[#]	22,049 (79)	1,788 (60)	250 (52)	51 (52)	31 (45)
**B Mutant (B^{M})**[#]	5,883 (21)	1,196 (40)	228 (48)	49 (49)	38 (55)
Heterozygots[#]	5,547 (20)	1,074 (36)	202 (42)	42 (42)	N.R.
Homozygots[#]	336 (1)	122 (4)	26 (5)	7 (7)	N.R.
T*		18,166,152	4,122,046	942,292	N.R.
Var(T)*		5.50E+11	7.64E+10	1.55E+10	N.R.
SD*		741,653	276,405	124,579	N.R.
Z*		24.4942	14.9131	7.5638	N.R.
p-value*		< 0.00001	< 0.00001	< 0.00001	< 0.00001
q-value[Δ]		0.000015	0.000015	0.000015	0.000015
RR[+]		2.25	3.33	3.58	4.57
OR[Φ]		2.51	3.42	3.60	4.59
		(2.41 – 2.61)	(3.12 – 3.75)	(2.95 – 4.40)	(3.61 – 5.85)
**AC^{M} and B^{M}**[#]	3,968 (14)	765 (26)	158 (33)	36 (36)	28 (41)
X^2[¶]		271.6949	134.4942	38.6564	39.1329
p-value[¶]		< 0.00001	< 0.00001	< 0.00001	< 0.00001
q-value[Δ]		0.000015	0.000015	0.000015	0.000015
RR[+]		1.91	2.91	3.38	4.10
OR[Φ]		2.08	2.98	3.40	4.12
		(1.99-2.18)	(2.70-3.29)	(2.76-4.19)	(3.23-5.27)
**AC^{M} and B^{WT}**[#]	6,362 (23)	518 (17)	75 (16)	17 (17)	10 (14)
X^2[¶]		45.7331	13.4671	1.8917	2.6871
p-value[¶]		< 0.00001	0.00024	0.16901	0.10116
q-value[Δ]		0.000015	0.00030	0.16901	0.10648
RR[+]		0.73	0.64	0.70	0.58
OR[Φ]		0.71	0.63	0.69	0.57
		(0.68-0.75)	(0.56-0.72)	(0.53-0.91)	(0.41-0.81)
**AC^{WT} and B^{M}**[#]	1,915 (7)	431 (14)	70 (15)	13 (13)	10 (14)
X^2[¶]		221.3469	43.8656	5.8729	6.2701
p-value[¶]		< 0.00001	< 0.00001	0.01538	0.01228
q-value[Δ]		0.000015	0.000015	0.01715	0.01449
RR[+]		2.06	2.28	2.02	2.30
OR[Φ]		2.29	2.32	2.03	2.30
		(2.17-2.44)	(2.08-2.70)	(1.51-2.74)	(1.63-3.24)
**AC_ and B_SNP Linkage Disequilibrium**					
X^2[¶]	2968.1236	358.0234	73.7101	16.1608	11.8413
p-value[¶]	< 0.00001	< 0.00001	< 0.00001	0.00006	0.00058
q-value[Δ]	0.000017	0.000017	0.000017	0.00008	0.00058
r^2	0.1063	0.1200	0.1542	0.1616	0.1716

[#] Data are presented as n or n (%). * Cochran–Armitage's trend test statistics (Armitage Test Statistic T (T), Variance of T (Var(T)), Standard Deviation (SD), Standard score (Z) and p-value). [Δ] q-value, Benjamini-Hochberg-adjusted p-value for False Discovery Rate (FDR) control. [+] RR, Relative Risk. [Φ] OR, Odds Ratio, 95% confidence interval in brackets. [¶] Pearson's chi-squared test statistics (Chi-squared (X^2) and p-value). N.R., not reported to avoid small cell counts in accordance with UK Biobank guidance.

SUPPLEMENTARY TABLE S2. Aggregated characteristics of UK Biobank doctor diagnosed idiopathic pulmonary fibrosis (IPF) participants.

A. Cohort summary

Characteristic	Value	Notes
Total participants	100	Caucasians, Whole genome sequence coverage > 95%
Age at diagnosis, years	65.0 (59.0-69.0)	Median (IQR)
BMI, kg/m^2	27.9 (25.5-31.3)	Median (IQR), calculated from available data
Weight, kg	81.4 (73.0-94.2)	Median (IQR), calculated from available data
Height, cm	170.0 (163.0-177.0)	Median (IQR), calculated from available data

B. Aggregated demographic, exposure, and underlying cause-of-death variables

Variable	Category	n (%)
Sex	Female	42 (42.0%)
	Male	58 (58.0%)
Age at diagnosis category	<=59 years	26 (26.0%)
	60-69 years	50 (50.0%)
	>=70 years	24 (24.0%)
BMI category	<25 kg/m2	22 (22.0%)
	25-<30 kg/m2	40 (40.0%)
	>=30 kg/m2	37 (37.0%)
Ever smoked	No	31 (31.0%)
	Yes	69 (69.0%)
Cigarettes/day	1-10 cigarettes/day	10 (10.0%)
	>10 cigarettes/day	37 (37.0%)
	Not reported	22 (22.0%)
Passive smoking	Rarely/never	39 (39.0%)
	Sometimes	42 (42.0%)
	Often	17 (17.0%)
Tobacco smoke exposure	Yes	87 (87.0%)
	No	12 (12.0%)
Underlying cause of death	No underlying cause of death recorded	59 (59.0%)
	J84.1 interstitial pulmonary diseases with fibrosis	20 (20.0%)
	Cancer-related causes	9 (9.0%)
	Other respiratory/cardiopulmonary causes	7 (7.0%)
	Other specified causes	5 (5.0%)

C. Selected variables by MUC5B promoter rs35705950 SNP carrier status

Variable	Category	MUC5B WT, n (%)	MUC5B mutant, n (%)
Sex	Female	27 (52.9%)	15 (30.6%)
	Male	24 (47.1%)	34 (69.4%)
Age group	<=59 years	19 (37.3%)	7 (14.3%)
	60-69 years	25 (49.0%)	25 (51.0%)
	>=70 years	7 (13.7%)	17 (34.7%)
BMI group	<25 kg/m2	10 (19.6%)	12 (24.5%)
	25-<30 kg/m2	20 (39.2%)	20 (40.8%)
	>=30 kg/m2	20 (39.2%)	17 (34.7%)
Ever smoked	No	19 (37.3%)	12 (24.5%)
	Yes	32 (62.7%)	37 (75.5%)
Cigarettes/day	>10 cigarettes/day	17 (33.3%)	20 (40.8%)
	Not reported	10 (19.6%)	12 (24.5%)
Passive smoking	Rarely/never	18 (35.3%)	21 (42.9%)
	Sometimes	24 (47.1%)	18 (36.7%)
	Often	7 (13.7%)	10 (20.4%)
Tobacco smoke exposure	Yes	42 (82.4%)	45 (91.8%)

D. Selected variables by MUC5AC rs878913005 SNP carrier status

Variable	Category	MUC5AC WT, n (%)	MUC5AC mutant, n (%)
Sex	Female	23 (48.9%)	19 (35.8%)
	Male	24 (51.1%)	34 (64.2%)
Age group	<=59 years	16 (34.0%)	10 (18.9%)
	60-69 years	18 (38.3%)	32 (60.4%)
	>=70 years	13 (27.7%)	11 (20.8%)

BMI group	<25 kg/m2	11 (23.4%)	11 (20.8%)
	25-<30 kg/m2	16 (34.0%)	24 (45.3%)
	>=30 kg/m2	19 (40.4%)	18 (34.0%)
Ever smoked	No	17 (36.2%)	14 (26.4%)
	Yes	30 (63.8%)	39 (73.6%)
Cigarettes/day	>10 cigarettes/day	14 (29.8%)	23 (43.4%)
	Not reported	10 (21.3%)	12 (22.6%)
Passive smoking	Rarely/never	17 (36.2%)	22 (41.5%)
	Sometimes	19 (40.4%)	23 (43.4%)
	Often	9 (19.1%)	8 (15.1%)
Tobacco smoke exposure	Yes	40 (85.1%)	47 (88.7%)

WT, wild type. Het, heterozygous. Hom, homozygous. BMI, body mass index. IQR, interquartile range.

SUPPLEMENTARY TABLE S3. Patient tissue used for immunostainings.

**Participant ID**	**Known lung disease**	**Smoking**	**Specimen type**	**MUC5AC** rs878913005 **C > T**	**MUC5B** rs35705950 **G > T**
1	IPF	No	Cryo-biopsy	N.A.	N.A.
2	IPF	No	Transplant	Homozygote	Heterozygote
3	IPF	No	Transplant	WT	WT
4	IPF	Previous	Transplant	Heterozygote	WT
5	IPF	Previous	Transplant	Heterozygote	WT
6	Adenocarcinoma	N.A.	Resection	WT	WT
7	Adenocarcinoma	No	Resection	Heterozygote	Heterozygote
8	Adenocarcinoma	Yes	Resection	Heterozygote	WT
9	Adenocarcinoma	N.A.	Resection	WT	WT
10	Healthy	Yes	Cryo-biopsy	N.A.	N.A.

All patients were WT for the Asian SNP (rs36189285) . N.A., not available.
